# Bibliographical

**Published:** 1863-05

**Authors:** 


### BIBLIOGRAPHICAL.

*Allport's Registering Dental Ledger. Published by S. S. White,* 528, *Arch street, Philadelphia.* 340 *pp.*

We have for several months been using a copy of this work, and know, by *experiment*, that it is an excellent thing. It is arranged for making a full registration by diagrams of all operations upon the teeth.

There are two diagrams upon each page, with rulings for the accounts of two persons, each ruling consisting of twenty-four lines. We have seen nothing better than this for the purpose; it is a complete register and account book combined.

It is an important matter to keep a correct and full register of all operations performed; and all special peculiarities marking any case should be carefully noted for further observation.

In regard to the style of the work, it is enough to say that it is published by S. S. White. T.

